# Treatment outcomes of pediatrics acute myeloid leukemia (AML) and associated factors in the country’s tertiary referral hospital, Ethiopia

**DOI:** 10.1186/s12885-024-12404-5

**Published:** 2024-05-24

**Authors:** Wudinesh Mamo, Ayalew Moges, Subah Abderehim Yesuf, Abdulkadir Mohamedsaid, Gashaw Arega

**Affiliations:** 1 Pediatrician and Child Health Specialist, Addis Ababa, Ethiopia; 2 Department of Pediatrics and Child Health, School of Medicine, Addis Ababa University, P.O. Box 9080, Addis Ababa, Ethiopia; 3 Department of Family Medicine, St. Peter Specialized Hospital, Addis Ababa, Ethiopia

**Keywords:** Paediatrics acute myeloid leukemia, Overall survival, Event-free survival, FAB subtype, Hyperleukocytosis

## Abstract

**Background:**

Pediatric Acute Myeloid Leukemia (AML) is a major cause of morbidity and mortality in children with cancer in Africa and other developing continents. Systemic chemotherapy and effective supportive care have significantly contributed to increased survival rates of pediatric AML in developed countries reaching approximately 70%. There is a paucity of contextual data regarding overall and event-free survival outcomes in children with acute myeloid leukemia in developing countries and most centers in Africa provide palliative care. The objective of this study was to assess the overall survival, event-free survival, and associated factors in pediatric AML patients treated in Ethiopia.

**Methods:**

This retrospective study was conducted on Pediatric AML patients treated at Tikur Anbessa Hospital between January 1, 2015, and May 30, 2022. The socio-demographic profile of patients, the clinical characteristics, the biochemical and morphological subtypes of AML were analyzed using SPSS version 25. The Kaplan–Meier survival curve was used to estimate the probabilities of overall and event-free survival. Statistical significance was set at *p* < 0.05.

**Results:**

A total of 92 children with AML were included in this study. The median age at diagnosis was 7 years (interquartile range: 5–10 years) with a slight male predominance. The median duration of symptoms was one month. Neutropenic fever (56, 86.2%) was the most common complication during treatment. About 29.3% of the patients succumbed to early death. The corresponding 1-year and 3-year OS probabilities were 28.2% and 23% respectively. The median event-free survival time for all pediatric AML patients was one-month (95% CI: 0.77–1.23). The determinants of poorer survival outcomes were FAB subtype, type of protocol used, and signs of CNS involvement (*p* < 0.05).

**Conclusion:**

The survival rates of children from AML were low in the study setting. More than 25% of AML patients succumbed to early death, and febrile neutropenia was the most common complication. Effective supportive and therapeutic measures should be taken to manage febrile neutropenia and to prevent early death in AML patients.

## Introduction

Acute Myeloid Leukemia comprises a heterogeneous group of hematologic malignancies characterized by the infiltration of the bone marrow and other tissues by abnormally proliferative myeloid precursors. It is much less common in children than acute lymphoblastic leukemia (ALL) and accounts for approximately 15% of childhood leukemias [1–3]. The global age-standardized incidence rate of leukemia is slightly higher in males than females (4, 5).

The cancer burden in Ethiopia may be significantly underestimated, mainly because of the lack of an organized national registry. AML is the second most common childhood leukemia diagnosed in the country, indicating that it is among the most common cancers occurring in the country, both in terms of incidence and mortality [4, 6, 11].

Most modern treatments for AML are multidisciplinary, with chemotherapy being the center of management [7, 19, 20]. Although significant improvements in outcomes have been achieved over the past decades, AML remains a life-threatening malignancy in children with a poor prognosis. In resource-rich countries, aggressive therapy, including the use of allogeneic hematopoietic cell transplantation (HCT), along with advanced supportive care has increased the survival rate for AML to nearly 70% , [2, 12] Unraveling the heterogeneity of the disease at the clinical, cytogenetic, and molecular levels has contributed to better prognosis in children with AML [8, 16–18].

AML patients are at high risk for life-threatening complications such as hyperleukocytosis, infections, hemorrhage, and typhlitis. Supportive care such as; hydration, antimicrobial prophylaxis, blood product transfusion, systemic antibacterial, and administering granulocyte colony-stimulating factor (G-CSF) during febrile neutropenia plays a significant role in the improvement of survival of pediatric AML patients [18, 20, 21].

In a study conducted in India, the median event-free survival (EFS) and overall survival (OS) were 12.6 and 14.6 months, respectively [15, 21]. In a study conducted in Brazil, OS) and EFS in children with AML-M3 were 69.2% and 67.7%, respectively, whereas, in other AML (other than AML-M3) patients, the corresponding rates were 45.3% and 36.7%, respectively [22].

In resource-challenged countries such as Ethiopia, it is presumed that the outcome might be low on the background of late patient presentations, lack of access to advanced diagnostic modalities, and supportive care [7, 9]. Van Weelderen et al. demonstrated that survival of AML among the Kenyan pediatric population was dismal and far lower than that of industrialized settings, with 2-year probabilities of event-free survival and overall survival of 4% and 7%, respectively [10]. In a study conducted in Egypt on 46 patients with AML showed that 54.3%) achieved complete remission, 21.7% had relapsed, and 69.6% were died [23]. The achievement of MRD negativity is associated with superior disease-free survival and overall survival in AML patients [24].

The absence of an effective cancer registry, national algorithms for AML reporting, and electronic data management systems in Ethiopia has made it challenging to obtain accurate population-based data on the prevalence and outcomes of patients with AML in the country. Moreover, the management of hematologic malignancies in general, and AML in particular, in low-income countries is suboptimal, partly because of the lack of advanced cytogenetic evaluation and supportive care [13–15].

This study was conducted to assess the survival of children diagnosed with AML and determine the contributing factors in pediatric patients attending a tertiary cancer center. No previous studies were done regarding the clinical profile and treatment outcome of childhood AML in Ethiopia. Therefore, this study will be the first to provide information in this regard and the results of this study will serve as a baseline to design strategies to improve the survival and quality of life of children with AML.

## Methods

### Study setting

The study was conducted in Tikur Anbessa Specialized Hospital, Addis Ababa, Ethiopia at the Pediatric Hematology and Oncology Unit, Addis Ababa, Ethiopia. Tikur Anbessa Specialized Hospital is the largest tertiary hospital in the country and was the only pediatric haemato-oncology treatment center in the county until recently. The pediatric haemato-oncology ward has 26 inpatient beds dedicated to pediatric Acute Leukemia/Lymphoma patients and the unit gives inpatient and outpatient clinic services to an estimated 8,000–10,000 children annually.

### Sampling

All children aged less than 15 years who were diagnosed with AML between January 1, 2015, and May 30, 2022, were included. A total of ninety-five (*n* = 92) met the inclusion criteria and were included in the study. Data were collected from July 1, 2022, to September 30, 2022.

### Data collection and data analysis

Data were collected by the principal investigator and trained general practitioners. The study questionnaires had four parts: Part I was about the socio-demographic profile of patients, Part II was about the clinical characteristics of patients at presentation, Part III was about the biochemical and morphological subtypes of AML, and Part IV was about the treatment profiles and survival outcomes of pediatric AML patients treated at Tikur Anbessa Specialized Hospital. The diagnosis of AML was suspected upon full blood count, and the diagnosis was confirmed by morphological examination of peripheral smear and bone marrow aspirates. For 12 (13%) patients, an additional flow cytometry was performed to establish a diagnosis.

After selecting the study cases, the data was collected from the registration log book, the patient card, and the follow-up chart by the data collectors. Data was entered into Epi data version 3.1 and exported to SPSS version 25 for analysis. Statistical significance was set at *P* < 0.05. Kaplan–Meier survival estimates were used for overall and event-free survival analyses.

### Ethical approval

Ethical approval was obtained from the Research and Ethics Committee of the Pediatrics and Child Health Department (DRCP), School of Medicine, College of Health Sciences, Addis Ababa University, and the College Institutional Review Board (IRB). Confidentiality was fully maintained during data collection and analysis. Participants were anonymous during the dissemination of the results.

## Results

### Sociodemographic characteristics

A total of ninety-two patients met the study inclusion criteria. The median age at diagnosis was 7 years (interquartile range: 5–10 years). A majority (39; 42.2%) of the patients were between 5 years to 10 years. Males constituted 59.6% (*n* = 55) with a male: female ratio of 1.5:1. Approximately half (45, 48.9%) of the patients were referred from a tertiary public hospital, whereas nearly a third (29;31.5%) were referred from a secondary public hospital. For the study population, the median time interval required to make a diagnosis from the day of admission was 10 days, with an IQR of 7.25–16.75. The corresponding time elapsed between presentation and the start of treatment was 16.5 (10.25–23.0) days (Table 1).

**Table 1:** Distribution by socio-demographic characteristics of pediatric acute myeloid leukemia patients at Tikur Anbessa Specialized Hospital, Addis Ababa, Ethiopia, 2015–2022 (*n* = 92)

Variable	Frequency	Percent (%)
**Age category**		
< 2 years	7	7.6
2–4.9 years	15	16.3
5–9.9 years	39	42.2
≥ 10 years	31	33.7
**Sex**		
Male	55	59.8
Female	37	30.2
**Residence**		
Oromia	32	34.7
Addis Ababa	20	21.7
SNNPR	16	17.4
Amhara	12	13.0
Other	12	13.0
**Year of diagnosis**		
2015	2	2.2
2016	3	3.3
2017	17	18.5
2018	13	14.1
2019	9	9.8
2020	16	17.4
2021	20	21.7
2022	12	13.0
**Source of referral**		
Private-public hospital	3	3.3
Secondary public hospital	29	31.5
Tertiary public hospital	45	48.9
Private facility	15	16.3
**Time interval before diagnosis in days (median + IQR)**	10	7.25–16.75
**Time interval before treatment in days (median + IQR)**	16.5	10.25–23.0

SNNPR: Southern Nations, Nationalities, and People’s Region; IQR: Interquartile range

### Clinical characteristics

Most (73;79.3%) of the patients presented to the hospital with symptoms that lasted for at least a month duration. Fatigue was the most common presenting complaint (*n* = 62, 67.4%), followed by fever and bleeding, which accounted for 50 (54.3%) and 41 (44.6%) of the patients, respectively.

Moreover, hepatomegaly, splenomegaly, and lymphadenopathy were the most commonly observed physical findings, presented in 58.7, 41.3 and 30.2% of patients, respectively. While a majority (62; 67.4%) of the patients had normal anthropometric parameters, moderate acute malnutrition, and severe acute malnutrition accounted for 16.3% (*n* = 15) of patients. Only ten (10.9%) patients had a pre-existing medical condition such as Down syndrome. Central nervous system (CNS) involvement was documented in 11 (16.4%) of the patients in whom CNS was assessed (Table 2).

**Table 2:** Clinical characteristics of pediatric acute myeloid leukemia patients at Tikur Anbessa Specialized Hospital, Addis Ababa, Ethiopia, 2015–2022 (*n* = 92)

Variable	Frequency	Percent (%)
**Duration of symptoms in months (median + IQR)**	1	1–2
**Presenting symptoms**		
Fever	50	54.3
Bleeding	41	44.6
Fatigue	62	67.4
Eye protrusion	6	6.5
Visual impairment	2	2.2
**Physical findings**		
Hepatomegaly	54	58.7
Splenomegaly	38	41.3
Lymphadenopathy	37	30.2
Gingival enlargement	10	10.9
Proptosis	8	8.7
Facial palsy	6	6.5
**Nutritional status**		
Normal	62	67.4
MAM	15	16.3
SAM	15	16.3
**Comorbidity**		
No	82	89.1
Yes	10	10.9
**Type of comorbidity** (*n* = 10)		
Down’s syndrome	6	60.0
Malaria	2	20.0
HIV/AIDS	1	10.0
Cardiac pathology^€^	1	10.0
**CNS involvement ** (*n* = 67)		
No	56	83.6
Yes	11	16.4

MAM: Moderate acute malnutrition; SAM: Severe acute malnutrition; CNS: Central nervous system; IQR: Interquartile range; ^€^ The cardiac pathology diagnosed is pre-existing, and not an iatrogenic cardiomyopathy

### Biochemical characteristics

Patient demographic data, laboratory tests, imaging studies, and pathological findings were obtained from medical records. Patient demographic data included age, and sex. The collected clinical and pathological features included Hashimoto’s thyroiditis (HT), ETE, tumour diameter, central lymph node metastasis (CLNM), lateral lymph node metastasis (LLCM), distant metastasis, total lymph node (LN) harvesting, total LN involvement and radioactive iodine therapy (RAI) after surgery.

The most common morphological subtype as per the FАB classification was АML M2, which was identified in 29 (31.5%) of the cases, followed by FAB-M4, noted in 21 (22.8%) of the patients. The baseline leukocyte count was less than 100*10^9^/L in seventy-one (77.2%) of the patients, with an overall median granulocyte count of 2550 (IQR: 994–22,650). The mean hemoglobin level at the time of diagnosis was 7.05 ± 2.56 g/dL. The median and IQR of baseline platelets in pediatric patients with AML were 24 × 10^9^/L) and 12–42.8, respectively. The median blast percentage was 59.5, with a range of 33.5–80.0% (Table 3).

**Table 3:** Biochemical characteristics of pediatric acute myeloid leukemia patients at Tikur Anbessa Specialized Hospital, Addis Ababa, Ethiopia, 2015–2022 (*n* = 92)

Variable	Frequency	Percent (%)
**Mode of diagnosis**		
Peripheral morphology (PM) alone	3	3.3
Bone marrow aspirate (BMA) alone	0	0
PM + BMA	76	82.6
PM + BMA + Flow cytometry	12	13.0
Flow cytometry	1	1.1
**FAB subtype**		
FAB-M0	4	4.3
FAB-M1	16	17.4
FAB-M2	29	31.5
FAB-M3	13	14.1
FAB-M4	21	22.8
FAB-M5	8	8.7
FAB-M7	1	1.1
WBC (*10 ^**9**^ **/L) (median + IQR)**	25.1	9.1–96.5
< 100	71	77.2
≥ 100	21	22.8
**ANC count**	2550	994–22,650
Platelets (*10 ^**9**^ **/L) (median + IQR)**	24	12–42.8
**Hemoglobin (g/dL) (mean ± SD)**	7.05	2.56
**Blast percentage (median + range)**	59.5	33.5–80.0

ANC: Absolute neutrophil count; FAB: French, American, and British; SD: Standard deviation; IQR: Interquartile range; WBC: White blood cell count

### Management courses

Treatment was initiated in 71 (77.2%) of the patients, and treatment was not initiated in the remaining 21 patients; the most frequent reasons for not initiating treatment were early death and leaving against medical advice, which accounted for 13 (61.9%) and 7 (33.7%) respectively.

Seven-plus-three (7 + 3 AML) protocol was the common type of therapeutic regimen started in the majority (*n* = 48;67.6%) of the patients. Completion of Chemotherapy was observed in 33 (46.5%) of the patients. Neutropenic fever (56, 86.2%) and tumor lysis syndrome (4, 6.2%) were the common complications during treatment. Complete remission was observed in 64.2% (*n* = 36) of the patients (Table 4).

**Table 4:** Management-related characteristics of pediatric acute myeloid leukemia patients at Tikur Anbessa Specialized Hospital, Addis Ababa, Ethiopia, 2015–2022 (*n* = 92)

Variable	Frequency	Percent (%)
**Treatment initiated**		
No	21	22.8
Yes	71	77.2
**Therapeutic intent** (*n* = 71)		
Curative	60	84.5
Palliative	8	11.3
Curative followed by palliative	3	4.2
**Reason for not initiating treatment** (*n* = 21)		
Death	13	61.9
Left against medical advice	7	33.3
Referred abroad	1	1.1
**Treatment completed** (*n* = 71)		
No	38	53.5
Yes	33	46.5
**Reason for not completing treatment** (*n* = 38)		
Death	24	63.2
Lost to follow up	7	18.4
Left against medical advice	7	18.4
**Type of protocol (71)**		
7 + 3 AML protocol	48	67.6
APML protocol	7	9.8
ADE protocol	8	11.2
AML Palliative	8	11.2
(**Complications during treatment** (*n* = 65)		
Neutropenic fever	56	86.2
Tumor lysis syndrome	4	6.2
Typhlitis	3	4.6
**Post-induction outcome** (*n* = 56)		
Complete remission	36	64.2
Failed remission	9	16.1
Death	11	19.6

APML: Acute promyelocytic leukemia; ADE: Cytarabine, daunorubicin, and etoposide ANC: Absolute neutrophil count; FAB: French, American, and British; SD: Standard deviation; IQR: Interquartile range; WBC: White blood cell count

### Survival outcomes

**Fig. 1:**
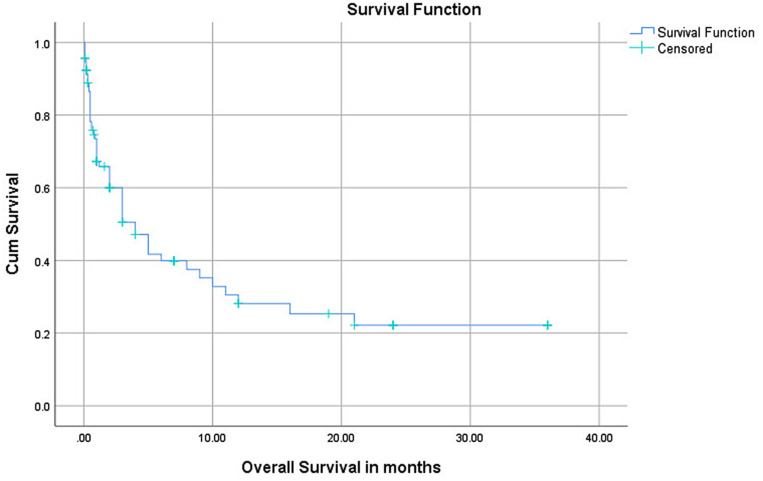
Overall survival outcomes of pediatric patients with acute myeloid leukemia at Tikur Anbessa Specialized Hospital, Addis Ababa, Ethiopia, 2015–2022 (*n* = 92)

**Fig. 2:**
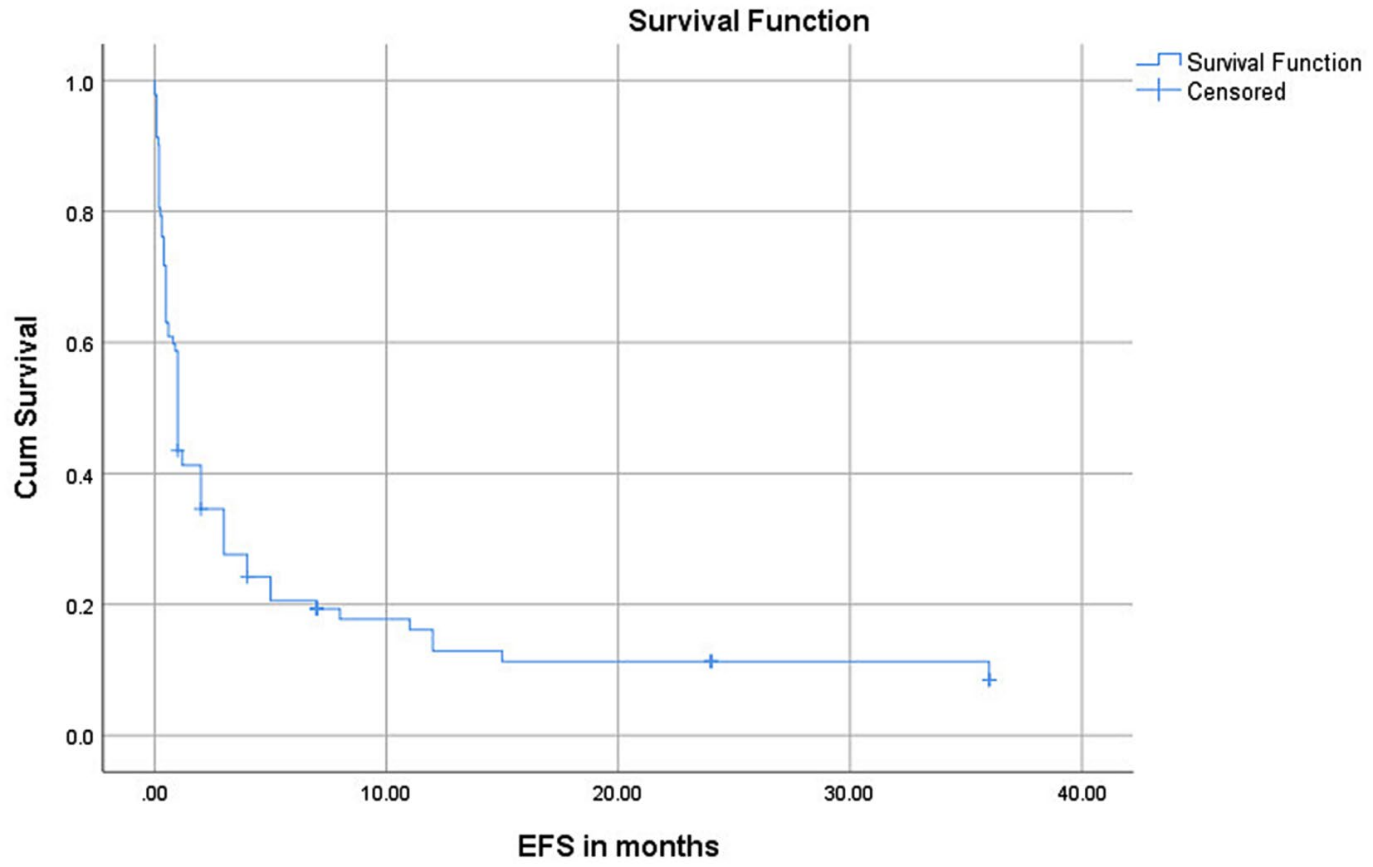
Event-free survival outcomes of pediatric patients with acute myeloid leukemia at Tikur Anbessa Specialized Hospital, Addis Ababa, Ethiopia, 2015–2022 (*n* = 92) EFS: Event-free survival

The median overall survival time for all pediatric AML patients was 4 months (95% CI: 2.10–5.90). Similarly, the median event-free survival time for all pediatric AML patients was one-month (95% CI: 0.77–1.23). Twenty-seven (29.3%) of the patients succumbed to early death, dying within the first six weeks of diagnosis. The corresponding 1-year EFS and OS probabilities for all pediatric AML patients were 16.1% and 28.2%, respectively. The third-year OS probability was 23%. (Figs. 1 and 2).

**Fig. 3:**
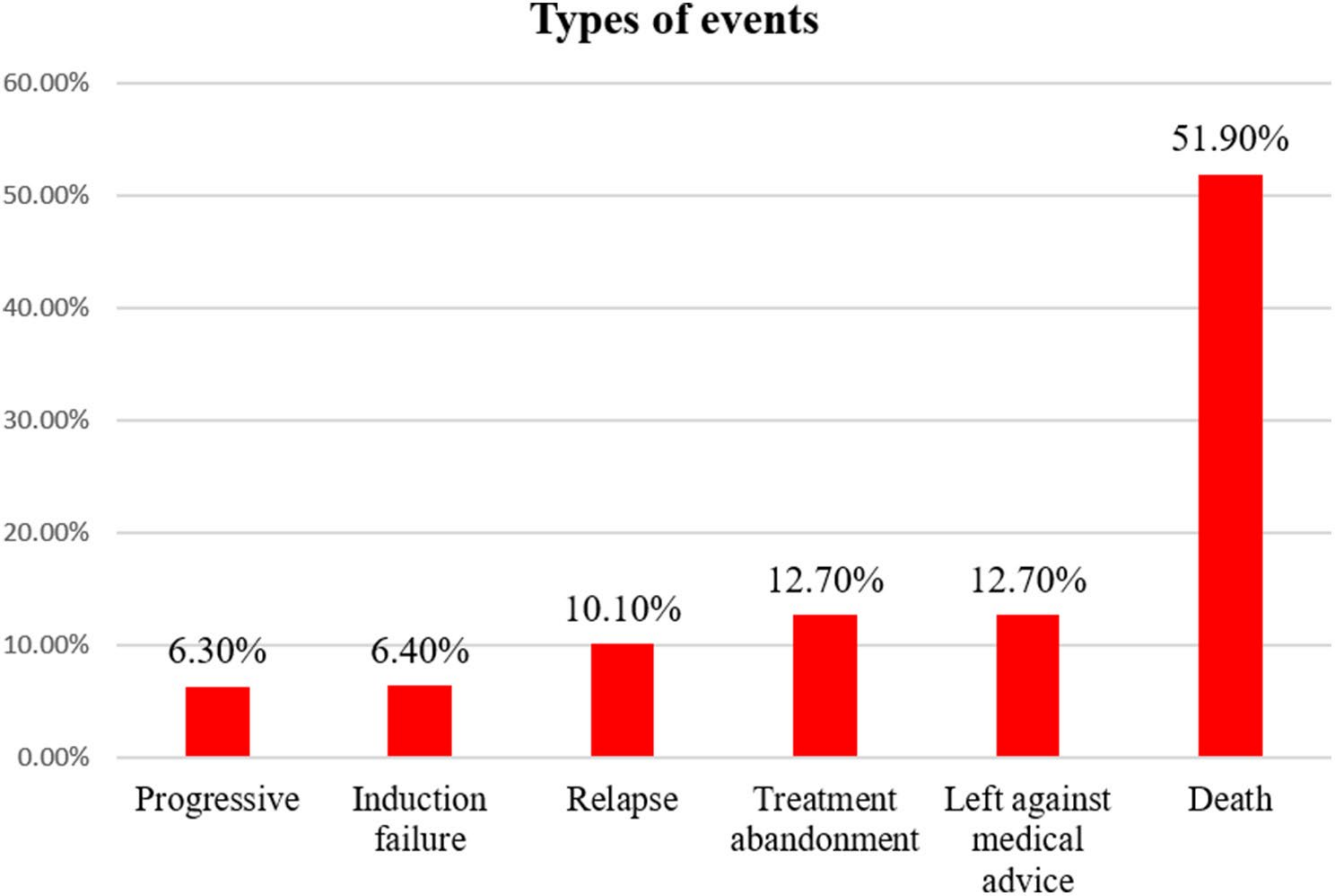
First events among pediatric acute myeloid leukemia patients at Tikur Anbessa Specialized Hospital, Addis Ababa, Ethiopia, 2015–2022 (*n* = 79)

In our study, the most frequent first event documented was death, occurring in forty-one (51.9%) of the patients, followed by treatment abandonment and leaving against medical advice. In contrast, disease progression occurred in 6.3% of the patients sustaining events (Fig. 3).

### Factors associated with survival outcomes

FAB subtype was shown to affect survival outcomes among the children with AML, with patients having FAB-M2 (3.0 months) showing better overall survival profiles than those having either FAB-M0 (0.6 months) or FAB-M7 (0.3 months). CNS involvement at the time of diagnosis was demonstrated to affect survival outcomes among children with AML. Patients with no CNS involvement had higher median survival rates than those with confirmed or undocumented CNS involvement. Patients who completed the treatment had a higher median overall and event-free survival rate than those who failed to complete the treatment. According to the log-rank test, factors such as age, sex, residence, and comorbidity did not have a statistically significant effect on survival outcomes among pediatric AML patients in the TASH (Table 5).

**Table 5:** Survival estimates according to sociodemographic and clinical characteristics of pediatric patients with AML at Tikur Anbessa Specialized Hospital, Addis Ababa, Ethiopia, 2015–2022

Variable	Event-free survival		Overall survival	
	Median	*P* value (log-rank)	Median	*P* value (log-rank)
**Age category**				
< 10 years	0.60	0.18	4.00	0.87
≥ 10 years	1.00		3.00	
**Sex**				
Male	1.00	0.56	5.00	0.12
Female	0.66		2.00	
**Residence**				
Urban	0.50	0.64	5.00	0.71
Rural	1.00		3.00	
**FAB subtype**				
FAB-M0	0.50	0.018	0.60	**0.002**
FAB-M1	0.50		2.00	
FAB-M2	1.00		6.00	
FAB-M3	4.00		-	
FAB-M4	2.00		3.00	
FAB-M5	0.50		4.00	
FAB-M7	0.30		0.30	
**Comorbidity**				
No	1.00	0.59	5.00	0.64
Yes	1.00		3.00	
**CNS involvement**				
No	1.20	**0.004**	6.00	**< 0.001**
Yes	1.00		5.00	
Undetermined	0.50		0.90	
WBC (*10 ^**9**^ **/L)**				
< 100	1.00	0.417 **<0.001** **0.016**	4.00	0.28 **< 0.001** **0.132**
≥ 100 **Treatment completed** NO YES **Type of protocol** 7 + 3 protocol APML protocol ADE protocol	0.50 1.2 7.0 3 - 8		3.00 2 21 9 - 8	

CNS: Central nervous system; FAB: French, American and British

## Discussion

Acute myeloid leukemia (AML) represents a treatment challenge for pediatric hemato-oncologists, particularly in low-resource settings (25, 26). This study was conducted to investigate the survival outcomes of patients with AML, along with the factors affecting the outcome in the Ethiopian context. Consequently, low event-free and overall survival outcomes were observed in the study population, along with certain factors such as the FAB subtype and signs of CNS involvement contributing to lesser survival outcomes.

Our study showed that the overall and event-free survival rates were low, with corresponding median survival outcomes of four months and one month, respectively. These one-year survival rates reported in the study (16.1% for EFS and 28.2% for OS) were better than those studies conducted in Tanzania and Western Kenya [10, 27]. However, these survival probabilities are low compared to those in other countries, such as Vietnam, Brazil, Mexico, and other developed countries [22, 25–31].

The poor survival outcome of patients with AML in the present study can be explained by late presentation, lack of diagnostic facilities, blood products, and unfavorable attitudes of clinicians in the study setting [9]. Additionally, the lack of optimal supportive care measures, such as unavailability of adequate antibiotics in febrile neutropenic patients, absence of air-conditioned one-person isolation rooms, poor hygiene within the wards, and increased nosocomial infections, as demonstrated in other similar settings, could have contributed to the worse outcome [7, 10].

The substantially inferior treatment outcomes in our setup can be primarily attributed to high abandonment, early death (ED), treatment-related mortality rates, and low recovery rates after relapse in the study setting, which commonly result from compromised supportive care and limited capacity, including unavailability of allogeneic stem cell transplantation [26]. In addition, the variation in survival probabilities across the study settings might have resulted from differences in cytogenetic profiles and pathohistological characteristics of the malignancies [32, 33, 36, 37].

The FAB subtype can predict the outcome of patients with AML, with FAB-M2 and FAB-M3 showing relatively better outcomes. The median survival time for the FAB-M3 category was not calculated as less than 50% of events (deaths) occurred by the end of the study period [33, 34]. However, this is in contrast to the report by Walter et al., in which the morphological subclassification of AML did not show significant prognostic information for cases of AML [35].

Furthermore, central nervous system involvement is associated with inferior survival, and our study also showed poorer outcomes in AML patients with CNS involvement [41–44].

## Conclusion

The median age at AML diagnosis in the children was 7 years, with an interquartile range of 5–10 years. Factors such as the FAB subtype, hyperleukocytosis, and signs of CNS involvement have been shown to shorten survival outcomes among children with AML in the study setting. Survival outcomes among pediatric patients with acute myeloid leukemia were low. Ongoing improvements will require mechanisms to control infection, timely therapeutic interventions, and effective supportive care measures to improve the survival and quality of life of children with AML.

### Strengths and limitations of the study

This study was conducted in one of the largest tertiary referral hospitals, serving as the only center for pediatric cancer patients until recently. We report the treatment outcomes of Acute Myeloid Leukemia in children, and this will serve as baseline data in the country; the study also gave a detailed complication for early detection for centers treating AML with curative intent.

Future research should identify barriers and psychosocial issues for loss to follow-up and abandonment of treatment.

## Data Availability

The datasets used and analyzed are available upon reasonable request from the corresponding author.
